# High‐Performance Polymer Solar Cells Based on a Wide‐Bandgap Polymer Containing Pyrrolo[3,4‐*f*]benzotriazole‐5,7‐dione with a Power Conversion Efficiency of 8.63%

**DOI:** 10.1002/advs.201600032

**Published:** 2016-04-25

**Authors:** Liuyuan Lan, Zhiming Chen, Qin Hu, Lei Ying, Rui Zhu, Feng Liu, Thomas P. Russell, Fei Huang, Yong Cao

**Affiliations:** ^1^ Institute of Polymer Optoelectronic Materials and DevicesState Key Laboratory of Luminescent Materials and DevicesSouth China University of TechnologyGuangzhou510640P.R. China; ^2^ State Key Laboratory for Artificial Microstructure and Mesoscopic PhysicsSchool of PhysicsPeking UniversityBeijing100871P.R. China; ^3^ Materials Sciences DivisionLawrence Berkeley National LaboratoryBerkeleyCA94720USA

**Keywords:** polymer solar cells, pyrrolo[3,4‐*f*]benzotriazole‐5,7‐dione, wide‐bandgap polymer

## Abstract

A novel donor–acceptor type conjugated polymer based on a building block of 4,8‐di(thien‐2‐yl)**‐**6‐octyl‐2‐octyl‐5*H‐*pyrrolo[3,4‐*f*]benzotriazole‐5,7(6*H*)‐dione (TZBI) as the acceptor unit and 4,8‐bis(5‐(2‐ethylhexyl)thiophen‐2‐yl)­benzo­[1,2‐*b*:4,5‐*b′*]dithiophene as the donor unit, named as PTZBIBDT, is developed and used as an electron‐donating material in bulk‐heterojunction polymer solar cells. The resulting copolymer exhibits a wide bandgap of 1.81 eV along with relatively deep highest occupied molecular orbital energy level of −5.34 eV. Based on the optimized processing conditions, including thermal annealing, and the use of a water/alcohol cathode interlayer, the single‐junction polymer solar cell based on PTZBIBDT:PC_71_BM ([6,6]‐phenyl‐C_71_‐butyric acid methyl ester) blend film affords a power conversion efficiency of 8.63% with an open‐circuit voltage of 0.87 V, a short circuit current of 13.50 mA cm^−2^, and a fill factor of 73.95%, which is among the highest values reported for wide‐bandgap polymers‐based single‐junction organic solar cells. The morphology studies on the PTZBIBDT:PC_71_BM blend film indicate that a fibrillar network can be formed and the extent of phase separation can be mani­pulated by thermal annealing. These results indicate that the TZBI unit is a very promising building block for the synthesis of wide‐bandgap polymers for high‐performance single‐junction and tandem (or multijunction) organic solar cells.

## 1 Introduction

Bulk‐heterojunction (BHJ) polymer solar cells (PSCs) have attracted considerable attention as a clean and renewable energy source due to the advantages of light‐weight, low‐cost solution processability, and flexible large‐area devices.^1^ Recent progress demonstrated high power conversion efficiencies (PCEs) larger than 10% for both single and multijunction PSCs.^2^ To access the commercialization, however, further improvement of the PCE is required, which can be achieved by developing efficient polymer donors for BHJ active layer materials. It is well established that the ideal electron‐donating conjugated polymers should have broad and strong absorption in both visible and near‐infrared regions to harvest solar radiation, appropriate highest occupied molecular orbital (HOMO) and lowest unoccupied molecular orbital (LUMO) energy levels to match the fullerene acceptor for efficient charge separation in the blend films, a high hole mobility for fast exciton diffusion and hole transport, and suitable compatibility with the fullerene acceptor to form a nanoscale bicontinuous network.[[qv: 1c]],^3^ These parameters can significantly affect the short‐circuit current density (*J*_sc_), open‐circuit voltage (*V*_oc_), and fill factor (FF) of PSCs. In device fabrication, various processing conditions, such as solvent additives, the usage of mixed solvents,^4^ thermal annealing,^5^ solvent annealing,^6^ and postannealing,^7^ are used to optimize the morphology of BHJ blends. In order to enhance the absorption over 800 nm to improve *J*_sc_, much effort has been dedicated to the development of low‐bandgap (LBG) conjugated polymers, typically adopting an alternating donor–acceptor (D–A) repeating unit strategy to tune the bandgap and energy levels by controlling the intramolecular charge transfer (ICT) from the donor (D) to the acceptor (A) moieties.[[qv: 1c]] Despite a variety of D–A type of LBG polymers have been developed toward high performance PSCs,^8^ the PCEs were limited by the *V*_oc_ due to the high‐lying HOMO energy levels in these materials. In comparison to the molecular design of LBG materials, developing wide‐bandgap (WBG) materials to optimize *V*_oc_ and charge extraction in devices is an alternative strategy to enhance device performance. This can lead to new materials that are transparent, can be integrated into building materials and multijunction devices, and used as bottom cells in tandem devices, which offers a new route to further increase device efficiency.^9^ In this regard, it is of high importance to develop novel WBG polymers with excellent photovoltaic properties in addition to high performance LBG polymers for tandem‐junction PSCs.

**Scheme 1:**
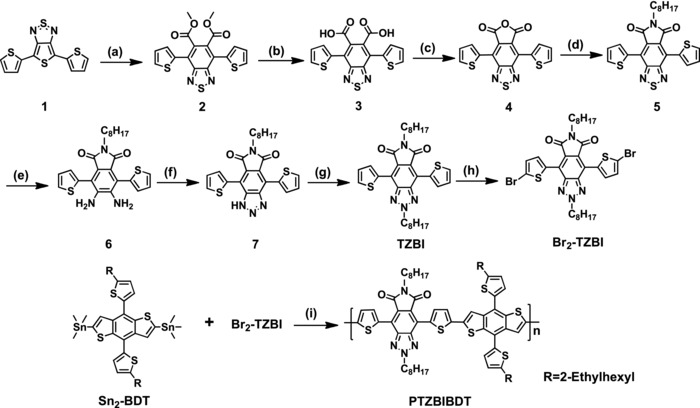
Synthetic routes for the monomer Br_2_‐TZBI and PTZBIBDT: a) dimethyl acetylenedicarboxylate, xylenes, reflux, 24 h; b) NaOH, EtOH, reflux, 24 h; c) Ac_2_O, xylenes, reflux, overnight; d) octylamine, AcOH, reflux, overnight, then, Ac_2_O, 110 °C, 8 h; e) Fe, AcOH, reflux, 5 h; f) NaNO_2_, THF, room temperature, 6 h; g) 1‐bromooctane, *tert*‐butoxide, MeOH, reflux, 24 h; h) NBS, CHCl_3_/AcOH, room temperature, 24 h; i) Pd(PPh_3_)_4_, toluene/DMF, reflux, 48 h.

WBG polymers can be synthesized by using moderate electron‐donating and electron‐withdrawing D–A pairs.^10^, ^11^, ^12^, ^13^, ^14^, ^15^, ^16^ For instance, Sun and co‐workers showed a successful example of poly{dithieno[2,3‐*d*:2′,3′‐*d′*]benzo[1,2‐*b*:4,5‐*b′*]dithiophene‐*co*‐1,3‐bis(thiophen‐2‐yl)‐benzo‐[1,2‐*c*:4,5‐*c′*]dithiophene‐4,8‐dione} (PDBT‐T1), which has a bandgap of 1.85 eV and a high PCE of 9.7%.[[qv: 10g]] Among the reported building blocks for WBG polymers, benzotriazole (BTA) unit has attracted much interest, since the lone electron pair on the 2‐position nitrogen atom reduces the electron–acceptor ability when compared to the thiadiazole‐based counterparts.[[qv: 10a,c]] It was also noted that the cyclic‐imide fused benzodiathiazole unit has recently been successfully employed as the electron‐accepting unit for the construction of low band‐gap conjugated polymers,^11^ since the cyclic imide can simultaneously reduce both LUMO and HOMO energy levels of the conjugated polymers. Inspired by the merits of the cyclic‐imide substituent in the conjugated backbone, we designed and synthesized a novel acceptor unit of cyclic‐imide substituted BTA, 4,8‐di(thien‐2‐yl)‐6‐octyl‐2‐octyl‐5*H‐*pyrrolo[3,4‐*f*]benzotriazole‐5,7(6*H*)‐dione (TZBI; **Scheme** **1**), for producing WBG polymers. By replacing the remaining two hydrogen atoms on the BTA unit with cyclic‐imide, both the HOMO and LUMO energy levels of the resulting polymer could decrease, which is beneficial to increase *V*_oc_ and PCE of PSCs.[[qv: 11b,c]] Furthermore, the accessibility of the N atom on the imide provides a straightforward route for alkylation to promote the solubility of the resulting polymers. To pair with TZBI, we chose benzo[1,2‐*b*:4,5‐*b′*]dithiophene (BDT)^12^ as a weak electron‐donating unit. BDT‐based conjugated polymers usually possess deep HOMO energy levels and high carrier mobilities, which is favorable for *V*_oc_ improvement. And thus we obtained a novel WBG copolymer PTZBIBDT. The detailed synthesis of monomer and polymer was shown in Scheme 1. The WBG copolymer PTZBIBDT exhibited optical bandgap (*E*_g_^opt^) of 1.81 eV. A remarkable photovoltaic performance with a PCE of nearly 7% was achieved for device using as‐cast active layers, which can be improved to 8.35% with thermal annealing, coming from increased *J*_sc_ and FF. Further device optimization using a water/alcohol polymer as cathode modification layer resulted in a PCE of 8.63%. To our knowledge, this is one of the highest PCE of donor–acceptor type conjugated polymers based on the benzotriazole derivatives and the short wavelength absorption that makes PTZBIBDT quite versatile in both single layer and multijunction devices.

## 2 Results and Discussion

The synthesis routes of monomer and copolymer are shown in Scheme 1, and their detailed synthesis procedures are available in the Supporting Information. The compounds of *N*‐octyl‐4,7‐di(thien‐2‐yl)‐5,6‐diamino‐isoindoline‐1,3‐dione (**6**) and 2,6‐bis(trimethyltin)‐4,8‐bis(5‐(2‐ethylhexyl)­thiophen‐2‐yl)benzo[1,2‐*b*:4,5‐*b′*]dithiophene (Sn_2_‐BDT) were prepared according to the previously reported method.^17^ 4,8‐Di(thien‐2‐yl)‐1H‐6‐octyl‐5*H‐*pyrrolo[3,4‐*f*]benzotriazole‐5,7(6*H*)‐dione (**7**) was obtained by the ring‐closing reaction of **6** with excessive NaNO_2_ in tetrahydrofuran (THF). The alkylation of **7** with 1‐bromooctane, in the presence of potassium *tert*‐butoxide, led to TZBI. Finally, the target compound Br_2_‐TZBI was synthesized through the bromination of compound TZBI by using *N*‐bromosuccinimide (NBS) in chloroform (CF).

The target copolymer PTZBIBDT was synthesized by palladium‐catalyzed Stille copolymerization of the dibromo‐monomer Br_2_‐TZBI and the bis(trimethyltin)‐monomer of Sn_2_‐BDT. The copolymer is readily soluble in common organic solvents such as CF, THF, toluene, and 1,2‐dichlorobenzene (*o*‐DCB). The number‐average molecular weight (*M*_n_) of PTZBIBDT is 22.5 kDa, with corresponding polydispersity index of 2.52, as determined by high temperature gel permeation chromatography at 150 °C in 1,2,4‐trichlorobenzene using polystyrene as the standard. The copolymer has excellent thermal stability with a decomposition temperature (defined as the 5% weight‐loss temperature, *T*_d_) higher than 340 °C (see Figure S1a in the Supporting Information), as evidenced by thermogravimetric analysis. However, no discernable glass transition temperature was observed by differential scanning calorimetry up to 300 °C (see Figure S1b in the Supporting Information).

**Figure 1:**
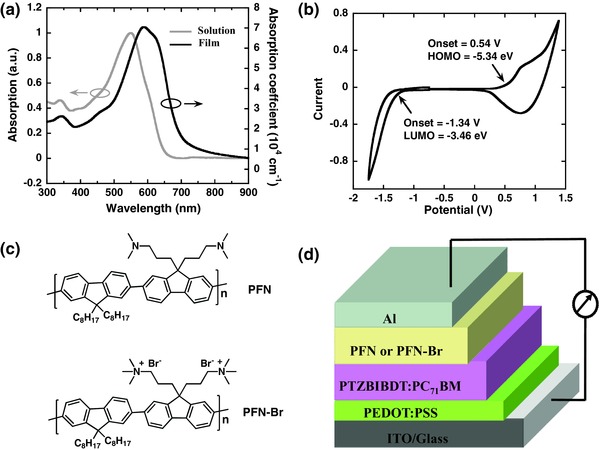
a) UV–vis absorption spectra of PTZBIBDT in diluted *o*‐DCB and thin film. b) CV curve of the polymer film on a platinum electrode measured in 0.1 mol L^−1^ Bu_4_NPF_6_ acetonitrile solutions at a scan rate of 50 mV s^−1^. c) Chemical structures of PFN and PFN‐Br. d) Device structure of organic solar cells used in this study.

The ultraviolet‐visible (UV–vis) absorption spectra of PTZBIBDT in diluted *o*‐DCB solutions and in thin films are shown in **Figure** **1**a. In solution, PTZBIBDT showed a major peak at 551 nm, which redshifted to 587 nm in the solid film due to strong intermolecular interactions. A distinct shoulder is seen at longer wavelengths in the film. In particular, PTZBIBDT exhibited a high absorption coefficient of 7.0 × 10^4^ cm^−1^. The optical bandgap (*E*_g_^opt^) of PTZBIBDT as estimated from the absorption onset in film is 1.81 eV. The frontier molecular orbital energy levels of the polymer were evaluated by electrochemical cyclic voltammetry (CV). As shown in Figure 1b, the onset of oxidation and reduction potentials referred to ferrocene/ferrocenium (Fc/Fc^+^) was determined to be 0.54 V and −1.34 V, corresponding to the HOMO energy level (*E*_HOMO_) and LUMO energy level (*E*_LUMO_) of −5.34 eV and −3.46 eV, respectively. Thus, the calculated electrochemical bandgap (*E*_g_^cv^) of PTZBIBDT was 1.88 eV, which was consistent with the optical bandgap. The low‐lying HOMO level of the polymer may ensure a high *V*_oc_ in solar‐cell devices since the *V*_oc_ is proportional to the offset between the HOMO level of donor material and the LUMO level of acceptor material.^18^

**Figure 2:**
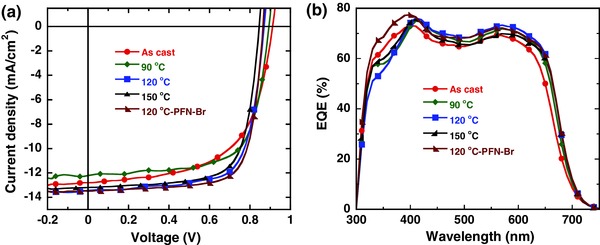
a) *J*–*V* characteristics and b) EQE curves of PSCs measured under AM 1.5G illumination (100 mW cm^−2^).

The photovoltaic performances of PTZBIBDT was investigated on the basis of the conventional PSCs with an architecture of ITO/PEDOT:PSS/PTZBIBDT:PC_71_BM/PFN/Al. Here, PC_71_BM is [6,6]‐phenyl‐C_71_‐butyric acid methyl ester. The photoactive layer was spin‐coated from *o*‐DCB solution. All devices were tested under AM 1.5G simulated illumination at 100 mW cm^−2^. The optimum ratio of PTZBIBDT:PC_71_BM was determined to be 1:1, and the optimum thickness of the photoactive layer was ≈80 nm. Device results are shown in **Figure** **2**a and **Table** **1**. Detailed device optimization is summarized in the Supporting Information. The as‐spun device showed an open circuit voltage (*V*_oc_) of 0.91 V, a short circuit current (*J*_sc_) of 12.80 mA cm^−2^, FF of 59.92%, and PCE of 6.98%. Thermal annealing was used to improve the device performance. After 15 min annealing at 90 °C the FF increased to 68.78%. However, *V*_oc_ and *J*_sc_ decreased slightly. An overall PCE of 7.48% was found. Increasing the annealing temperature to 120 °C further improved FF to 71.09%, and *J*_sc_ increased to 13.50 mA cm^−2^. A PCE of 8.35% was seen. When annealed at 150 °C, FF was improved to 71.80%; however *V*_oc_ and *J*_sc_ both decreased, reducing the PCE to 8.06%. Further optimization of device performance was carried out by using a conjugated polyelectrolyte interfacial layer of poly[(9,9‐bis(3‐(*N*,*N*‐dimethyl)‐*N*‐ethylammonium)‐propyl)‐2,7‐fluorene)‐*alt*‐2,7‐(9,9‐dioctylfluorene)]dibromide (PFN‐Br) for the 120 °C annealing condition.^19^ It was seen that FF of the device increased to 73.95% and a PCE of 8.63% was obtained. To our knowledge, a PCE of 8.63% is among the highest values reported for single‐junction PSCs based on WBG polymers.

**Table 1:** Photovoltaic performance of the devices based on PTZBIBDT:PC_71_ BM (1:1, wt:wt) under the illumination of AM1.5G, 100 mW cm^−2^

Device	*V*_oc_[V]	*J*_sc_[mA cm^–2^]	FF[%]	PCE[%]	AveragePCEb) [%]
As cast	0.91	12.80	59.92	6.98	6.68 ± 0.24
90 °C	0.89	12.22	68.78	7.48	7.28 ± 0.20
120 °C	0.87	13.50	71.09	8.35	8.27 ± 0.12
120 °Ca)	0.87	13.50	73.95	8.63	8.54 ± 0.14
150 °C	0.85	13.20	71.80	8.06	7.89 ± 0.12

^a)^Device using PFN‐Br cathode interlayer/Al;

^b)^The average PCE based on ten devices.

External quantum efficiency (EQE) spectra of the optimal devices are shown in Figure 2b. For all devices a broad EQE response in the range of 300–700 nm was recorded. The maximum value of EQE reached about 70%–80%. The calculated current densities from the integrated EQE spectra are inconsistent with those obtained from *J*–*V* measurements. It can be seen that thermal annealing led to an overall enhancement of the EQE across the full spectra region. The best EQE curve was obtained for film annealed at 120 °C. A slight decrease in the long wavelength region was seen for the film annealed at 150 °C. When PFN‐Br was used as the cathode modification interlayer, EQE in the 300–400 nm region was enhanced, but 600–700 nm was slightly lower than PFN devices. Thus, a similar *J*_sc_ was obtained.

The effects of thermal annealing on the charge carrier mobility of the polymer were investigated using hole‐only diodes based on the space charge limited current method (see Figure S5 in the Supporting Information). When the blend films were thermally annealed at 90, 120, and 150 °C, the measured hole mobility of PTZBIBDT was 1.89 × 10^−4^ cm^2^ V^−1^ s^−1^, 3.38 × 10^−4^ cm^2^ V^−1^ s^−1^, and 2.66 × 10^−4^ cm^2^ V^−1^ s^−1^, respectively. In particular, the hole mobility of PTZBIBDT was about 30 times larger than pristine film (9.67 × 10^−5^ cm^2^ V^−1^ s^−1^) after thermal annealing at 120 °C. A higher hole mobility is beneficial for the hole collection and reduces the competing nongeminate recombination,^20^ resulting in the enhanced *J*_sc_ and FF in device.

**Figure 3:**
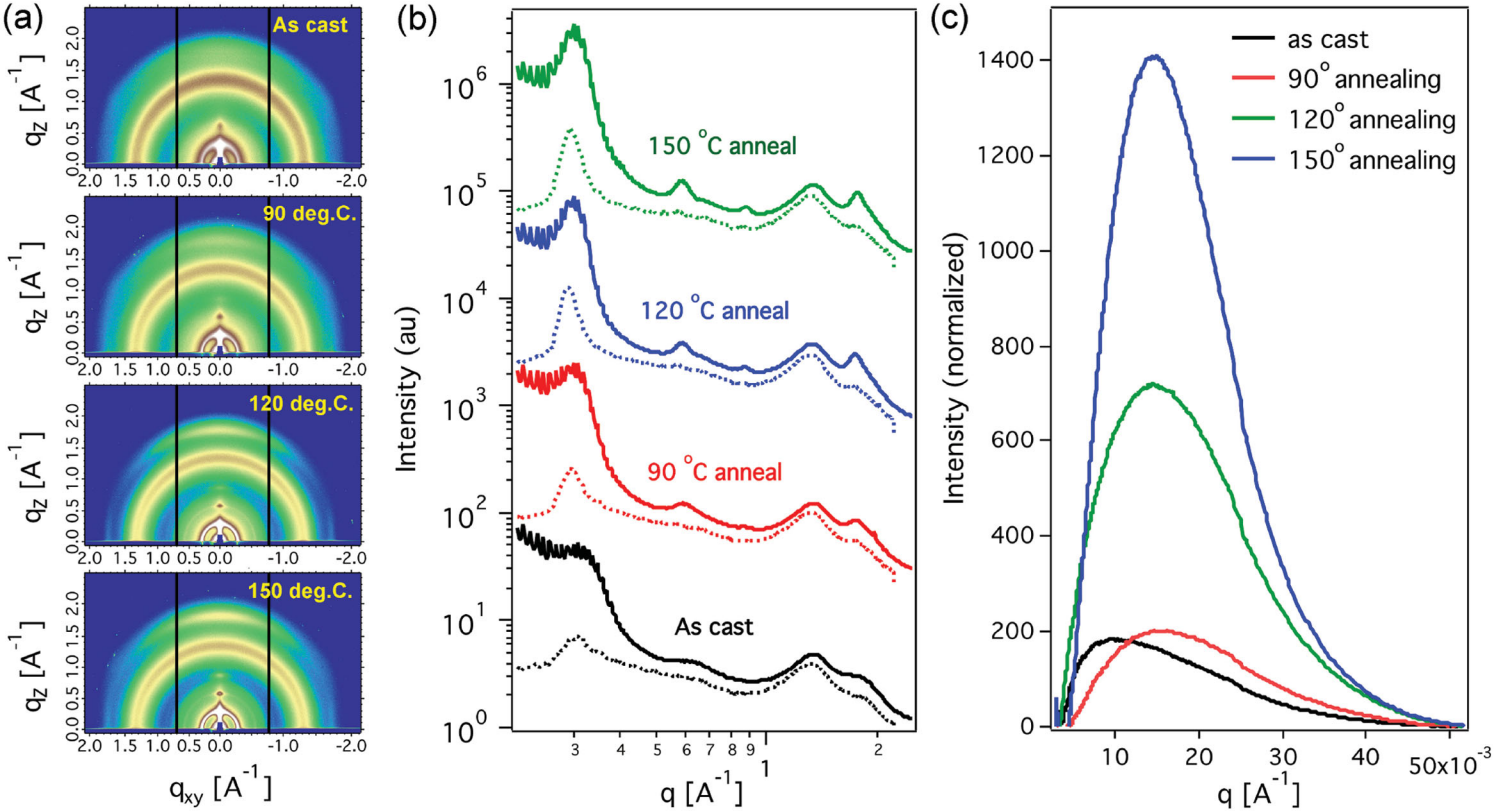
a) GIXD diffraction images of PTZBIBDT:PC_71_ BM BHJ thin film; b) line cut profiles (solid line: out‐of‐plane; dotted line: in‐plane) of GIXD results; c) RSoXS of PTZBIBDT:PC_71_BM blends processed from different annealing conditions.

The morphology of BHJ thin film played a critical role in the device performance. For the current system, the improved device performance after thermal annealing can be attributed to an optimized film morphology. The order of PTZBIBDT in BHJ blends was studied using grazing incidence X‐ray diffraction (GIXD). Shown in **Figure** **3**a,b are diffraction profiles and line‐cuts of the profiles. As seen from the diffraction pattern, a broad (100) peak is seen at 0.31 A^−1^, corresponding to a distance of 2.02 nm. PC_71_BM showed a distinctive peak at ≈1.35 A^−1^. The π–π stacking (010) reflection is quite weak for this sample. Thus, it was not surprising that a poor hole‐mobility was found. When the BHJ thin film was annealed at 90° for 15 min, PTZBIBDT showed improved crystallization. (100) peak became sharper and π–π stacking peak emerged at 1.77 A^−1^, corresponding to a distance of 0.35 nm. The coherence length of the crystal (CCL) along the (100) calculated from in‐plane direction was 17.2 nm; π–π stacking crystal coherence length calculated from out‐of‐plane direction was 1.6 nm. Annealing at 120 °C further enhanced the (100) and (010) peak intensity and CCL. A (100) CCL of 23.5 nm and a (010) CCL of 2.8 nm were found. By annealing at 150 °C, a slight reduction in the intensity of the (100) and the CCL was observed, while the (010) crystal peak was similar to that of the sample annealed at 120 °C.

**Figure 4:**
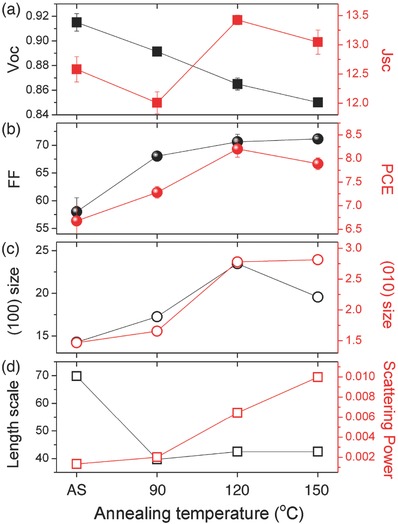
Summary of device performance and morphology versus different annealing temperature: a) open‐circuit voltage and short‐circuit current density; b) fill factor and power conversion efficiency; c) (100) crystal size and (010) crystal size of blended thin films; d) length scale and scattering power of blended thin films.

Resonant soft X‐ray scattering (RSoXS) was used to probe the length scale of phase separation in BHJ thin films.^21^ Shown in Figure 3c are the scattering profiles from thin films processed at different annealing temperatures. It is obvious that the as‐cast thin film showed a poorly defined scattering peak at ≈0.01 Å^−1^, corresponding to a distance of 62.8 nm. The scattering peak showed a log normal distribution and, therefore, a smaller length scale of phase separation existed in as‐cast sample. Thermal annealing at 90 °C led to a slight shift of scattering peak to 0.0147 Å^−1^, giving a distance of 42.7 nm. The scattering peak intensity increased very slightly, and, thus, no major changes in the morphology. Upon annealing at 120 °C, the scattering intensity increased strongly, revealing a major phase separation process at this temperature treatment. The length scale of phase separation remained at 42.7 nm. It should be noted that the (100) crystal size (CCL) estimated from in‐plane line cut profiles in GIXD gives a value of 23.5 nm, and the blending ratio of PTZBIBDT:PC_71_BM is 1:1. Thus the polymer crystallites in BHJ film give rise to the scattering. The 42.7 nm distance corresponds to the average crystal‐to‐crystal spacing or the mesh size in the thin film. Thus, thermal annealing induced a crystallization producing a network structure in the BHJ film. This was verified by transmission electron microscopy where a fibrillar network morphology (Figure S7 in the Supporting Information) was observed. The well‐developed sharp scattering reflection indicates the morphology is binary. The relative scattering power was estimated by integrating *Iq^2^* over all *q*, which reflects the extent of phase separation. Shown in **Figure** **4** are the device performances, morphologies, and annealing temperatures, suggesting a correlation. A decrease in *V*_oc_ was seen when the annealing temperature was increased, which can be attributed to enhanced polymer stacking in the blends. *J*_sc_ in the as‐cast thin film was slightly higher than 90 °C annealed sample; we suspect this is due to a broader size distribution of scatter. The as‐cast BHJ film is more of a multilength scale morphology. From 90 to 120 °C, the increased phase purity can be a major reason for *J*_sc_ improvement. However, this trend does not hold for even higher temperature processing. The sample annealed at 150 °C showed the highest contrast or phase purity, but the *J*_sc_ decreased in comparison to the 120 °C annealed sample. The fill factor correlated well with (010) crystal size and phase purity. This is easy to understand since FF is closely related to thin film electrical properties. A full spectrum analysis of the *J*_sc_ would be needed to take account of length scale of phase separation, domain distribution, and domain purity, and there is still no quantitative analytical method to evaluate this parameter.

## 3 Conclusion

In summary, we have designed and synthesized a novel donor–acceptor type wide bandgap conjugated copolymer PTZBIBDT based on TZBI as the acceptor and BDT as the donor unit for photovoltaic applications. The polymer exhibited a wide bandgap of 1.81 eV along with a low‐lying HOMO energy level of −5.34 eV. Conventional polymer solar cell with high PCE of near 7% was obtained based on PTZBIBDT:PC_71_BM without any post‐treatments. Further optimization of the solar cell device by using thermal annealing and water/alcohol interlayer resulted in a power conversion efficiency of 8.63%. To our knowledge, this is among the highest PCEs of D–A type conjugated polymers based on the benzotriazole derivatives as the electron‐deficient units for single‐junction PSCs and also the reported wide bandgap polymers. PTZBIBDT:PC_71_BM blends showed a fibrillar network morphology, and the extent of phase separation can be manipulated by thermal annealing. A detailed morphological investigation revealed the origin of performance improvement upon thermal treatment. These results indicate that TZBI unit is a promising building block for the construction of wide bandgap conjugated polymers for high performance single‐junction and tandem (or multijunction) organic solar cells.

## Supporting information

As a service to our authors and readers, this journal provides supporting information supplied by the authors. Such materials are peer reviewed and may be re‐organized for online delivery, but are not copy‐edited or typeset. Technical support issues arising from supporting information (other than missing files) should be addressed to the authors.

Supplementary
