# Supplementary material for: High‐Performance Polymer Solar Cells Based on a Wide‐Bandgap Polymer Containing Pyrrolo[3,4‐f]benzotriazole‐5,7‐dione with a Power Conversion Efficiency of 8.63%
Source: Adv Sci (Weinh). 2016 Apr 25;3(9):1600032. doi: 10.1002/advs.201600032 (PMC5039964; doi:10.1002/advs.201600032)
Supplement: Supplementary file 1 — Supplementary [file ADVS-3-0n-s001.pdf]

Copyright WILEY-VCH Verlag GmbH & Co. KGaA, 69469 Weinheim, Germany, 2016.

ADVANCED SCIENCE Open Access

Supporting Information

for *Adv. Sci.,* DOI: 10.1002/advs.201600032

High-Performance Polymer Solar Cells Based on a Wide-Bandgap Polymer Containing Pyrrolo[3,4-*f*]benzotriazole-5,7-dione with a Power Conversion Efficiency of 8.63%

Liuyuan Lan, Zhiming Chen, Qin Hu, Lei Ying, Rui Zhu, Feng Liu,* Thomas P. Russell,* Fei Huang,* and Yong Cao*

Copyright WILEY-VCH Verlag GmbH & Co. KGaA, 69469 Weinheim, Germany, 2013.

Supporting Information

High-Performance Polymer Solar cells Based on a Wide-Bandgap Polymer Containing Pyrrolo[3,4-*f*]benzotriazole-5,7-dione with a Power Conversion Efficiency of 8.63%

Liuyuan Lan, Zhiming Chen, Qin Hu, Lei Ying, Rui Zhu, Feng Liu*, Thomas P. Russell*, Fei Huang* and Yong Cao*

L. Y. Lan, Z. M. Chen, Prof. L. Ying, Prof. F. Huang, Prof. Y. Cao
Institute of Polymer Optoelectronic Materials and Devices
State Key Laboratory of Luminescent Materials and Devices
South China University of Technology
Guangzhou 510640, China
E-mail: msleiying@scut.edu.cn; msfhuang@scut.edu.cn

Dr. F. Liu, Prof. T. P. Russell
Materials Sciences Division, Lawrence Berkeley National Laboratory, Berkeley, California 94720, United States
E-mail: iamfengliu@gmail.com; russell@mail.pse.edu

Q. Hu, Prof. Rui Zhu
State Key Laboratory for Artificial Microstructure and Mesoscopic Physics, School of Physics, Peking University, Beijing, 100871, China

1. Experimental Section

Measurements. ^{1}H and ^{13}C NMR were recorded on a Bruker-400 spectrometer with tetramethylsilane (TMS) as the internal reference. The number-average molecular weights

(M_n), weight-average molecular weights (M_w) and polydispersity index (PDI) of copolymers were determined at 150 °C by a PL-GPC 220 type in 1,2,4-trichlorobenzene using a calibration curve with standard polystyrene as a reference. The differential scan calorimetry (DSC) were measured on a Netzsch DSC 204 under N_2 flow at a heating rate of 10 °C/min and cooling rate of 20 °C/min. Thermogravimetric analysis (TGA) was performed on Netzsch TG 209 in nitrogen, with a heating rate of 20 ºC min^{-1}. UV-vis absorption spectra were recorded on a HP 8453 spectrophotometer. Cyclic voltammograms measurements (CV) were recorded on a CHI 660A electrochemical workstation. The measurements were carried out in a nitrogen-saturated solution of 0.1 M tetra-*n*-butylammonium hexafluorophospate (*n*-Bu_4NPF_6) in acetonitrile with platinum electrode against Ag/AgCl referenc electrode. The scan rate was 50 mV s^{-1}. A platinum electrode coated with thin copolymer film was used as the working electrode. Tapping-mode atomic force microscopy (AFM) images were obtained using a NanoScope NS3A system (Digital Instrument) or a Bruker Multi-mode 8 system. Transmission electron microscope (TEM) images were characterized with a JEM-2100F instrument. The external quantum efficiency (EQE) data were recorded with a QE-R test system from Enli technology company (Taiwan).

Fabrication and characterization of PSCs. Patterned indium tin oxide (ITO)-glass substrates were used as the anode in the polymer solar cells. The ITO coated glass substrates were cleaned by sonication in detergent, deionized water, acetone and isopropyl alcohol; and then dried in a nitrogen stream, followed by an oxygen plasma treatment. Then the surface of the ITO substrate was modified by spin-coating the conducting poly(3,4-ethylenedioythiophene): poly(styrene sulfonic acid) (PEDOT:PSS) (Clevios P4083) layer with a thickness of 40 nm, followed by baking at 150 °C for 10 minutes under ambient conditions. The substrates were then transferred into an nitrogen-filled glove box. The copolymers were blended with $PC_{61}BM$ or $PC_{71}BM$ and dissolved in 1,2-dichlorobenzene (*o*-DCB). The solutions were then spin-coated onto the PEDOT:PSS layer at 800-1000 rpm. The

thicknesses of the active layer were about 80-90 nm. Thermal annealing of the blend films was carried out by placing them onto a hot plate with different temperatures for 15 minutes in a nitrogen atmosphere. A 5 nm PFN or PFN-Br layer was then spin-coated from methanol solution in presence of a trace amount of acetic acid onto the active layer. Subsequently, the films were transferred into a vacuum evaporator and 80 nm of Al were deposited as cathode. The effective area of a device was 0.04 cm^2 as determined by the shadow mask used during deposition of Al cathode.

The current-voltage (*J–V*) characteristics of photovoltaic devices were measured on a computer-controlled Keithley 2400 source meter under 1 sun, AM 1.5 G spectrum from a class solar simulator (Japan, SAN-EI, XES-40S1). The light intensity was 100 mW cm^{-2} as calibrated by an NREL certified reference monocrystal silicon cell (Hamamatsu).

Space Charge Limited Current (SCLC) measurement. Hole-only devices were fabricated to measure the hole mobility using the space charge limited current (SCLC) method with a device configuration of ITO/PEDOT:PSS/polymer:$PC_{71}BM$/MoO_3/Al. The PEDOT:PSS layer was spin-cast onto the pre-cleaned ITO glass followed by thermal annealing at 150 ^oC for 15 min under ambient conditions. The substrates were then transferred into an argon-filled glove-box. Subsequently, the BHJ composite films were prepared on ITO/PEDOT:PSS substrates using the same method as that for solar cell device fabrication. Finally, MoO_3 (~10 nm) and Al (~80 nm) were sequentially thermally deposited on the top of the active layer in a vacuum system. The mobility was determined by fitting the dark current to the model of a single carrier SCLC, described by the equation: $J_{SCLC} = (9/8)\varepsilon_0\varepsilon_r\mu_0(V^2/L^3)$, where J was the current, μ_0 is the zero-field mobility, ε_0 was the permittivity of free space, ε_r was the relative permittivity of the material, L was the thickness of the active layer, and V was the effective voltage. The effective voltage can be obtained by subtracting the built-in voltage (V_{bi}) and the voltage drop (V_s) from the substrate's series resistance from the applied voltage (V_{appl}), $V = V_{appl} - V_{bi} - V_s$. The hole-mobility can be calculated from the slope of the $J^{1/2}$–V curves.

GIXD characterization. Grazing incidence X-ray scattering characterization of the thin films was performed at the Advanced Light Source on beamline 7.3.3, Lawrence Berkeley National Lab (LBNL). Thin film samples were spin-casted on to PEDOT:PSS covered SiO_2 wafers. SiO_2 wafers were cleaned by sonication in detergent, deionized water, acetone and isopropyl alcohol. Then the surface of the SiO_2 wafers was modified by spin-coating a PEDOT:PSS layer with a thickness of 40 nm followed by drying at 120 ^{o}C for 30 min. After that, the BHJ films were spin-casted on SiO_2/PEDOT:PSS substrates under exactly the same conditions as those for the fabrication of solar cell devices. The scattering signal was recorded on a 2D detector (Pilatus 2M) with a pixel size of 0.172 mm by 0.172 mm. The samples were ≈15 mm long in the direction of the beam path, and the detector was located at a distance of ≈300 mm from the sample center (distance calibrated using a silver behenate standard). The incidence angle of 0.16° was chosen which gave the optimized signal-to-background ratio. The beam energy was 10 keV, operating at top-off mode. Typically, 30 s exposure time was used to collect diffraction signals. All GIXD experiments were done in helium atmosphere. The data was processed and analyzed using Nika software package.

RSoXS characterization. RSoXS was performed at beamline 11.0.1.2 Advanced Light Source, LBNL. Thin film samples were spin-casted on to PEDOT:PSS covered SiO_2 wafers. SiO_2 wafers were cleaned by sonication in detergent, deionized water, acetone and isopropyl alcohol. Then the surface of the SiO_2 wafers was modified by spin-coating a PEDOT:PSS layer with a thickness of 40 nm followed by drying at 120 ^{o}C for 30 min. After that, the BHJ films were spin-casted on SiO_2/PEDOT:PSS substrates under exactly the same conditions as those for the fabrication of solar cell devices. The scattering signals were collected in vacuum using Princeton Instrument PI-MTE CCD camera.

2. Experimental details

All chemicals and reagents were purchased from commercial sources (Aldrich, Acros,

and Alfa Aesar) and used without further purification unless stated otherwise. Toluene and tetrahydrofuran (THF) were purified by routine procedures and distilled in dry argon atmosphere before used. The compounds of *N*-octyl-4,7-di(thien-2-yl)-5,6-diamino-isoindoline-1,3-dione (**6**)[1] and 2,6-bis(trimethyltin)-4,8-bis(5-(2-ethylhexyl)thiophen-2-yl)benzo[1,2-*b*:4,5-*b'*]dithiophene (**Sn_2-BDT**)[2] were synthesized according to reported procedures.

4,8-Di(thien-2-yl)-1H-6-octyl-5H-pyrrolo[3,4-f]benzotriazole-5,7(6H)-dione (7)

Compound **6** (2 g, 4.4 mmol) was dissolved in the tetrahydrofuran at the room temperature. $NaNO_2$ (1.25 g, 18.24 mmol) was dissolved in water (20 mL) and was then added into the solution of compound **6** dropwise and the reaction was stirred for about 6 h. The mixture was extracted with CH_2Cl_2, the combined organics were dried with $MgSO_4$, filtered, and concentrated under vacuum to give product as a pale yellow solid (1.7 g, crude yield = 86%). The crude product was used directly in the next reaction without further purification.

*4,8-Di(thien-2-yl)-6-octyl-2-octyl-5H-pyrrolo[3,4-f]benzotriazole-5,7(6H)-dione (**TZBI**)*

Compound **7** (1.7 g, 3.66 mmol) was suspended in the methanol at the room temperature. Potassium *tert*-butoxide (0.5 g, 4.4 mmol) and 1-bromooctane (0.85 g, 4.4 mmol) were added under the protection of argon. The reaction was stirred under reflux condition for 24 h. The solvent was removed by evaporation to give the crude product, which was purified by the column chromatography on silica to give the target compound as a yellow solid (0.75 g, yield = 35.5%). ^{1}H NMR (400MHz, $CDCl_3$): δ=8.05 (dd, J_1 = 2.4 Hz, J_2 = 0.84 Hz, 2H), δ=7.64 (dd, J_1 = 3.4 Hz, J_2 = 0.76 Hz, 2H), δ=7.24 (t, J = 5.84 Hz, 2H), δ=4.75 (t, J = 9.72 Hz, 2H), δ=3.68 (t, J = 10.0 Hz, 2H), δ=2.12 (m, 2H), δ=1.66 (m, 2H), δ=1.26-1.29 (m, 20H), δ=0.83 (m, 6H). ^{13}C NMR (151 MHz, $CDCl_3$) δ 166.62, 145.93, 132.87, 132.01, 129.59, 126.86,

125.08, 123.87, 57.54, 38.53, 31.78, 31.71, 29.91, 29.19, 29.17, 29.07, 28.91, 28.39, 27.03, 26.49, 22.63, 22.61, 14.09.

*4,8-Di(5-bromothien-2-yl)-6-octyl-2-octyl-5H-pyrrolo[3,4-f]benzotriazole-5,7(6H)-dione (**Br_2-TZBI**)*

Compound **TZBI** (0.83 g, 1.44 mmol) was dissolved in chloroform, and then *N*-bromosuccinimide (NBS) (0.64 g, 3.6 mmol) was added in several portions. The mixture was stirred for 24 h at the room temperature. The mixture was extracted with CH_2Cl_2, the combined organics were dried with $MgSO_4$, filtered, and concentrated under vacuum. The crude product was purified by silica column chromatography and recrystallization from ethanol afforded the title compound as an orange solid (0.92 g, yield = 87.6%). ^{1}H NMR (400MHz, $CDCl_3$): δ=7.97 (d, J = 3.24 Hz, 2H), δ=7.19 (d, J = 3.2 Hz, 2H), δ=4.76 (t, J = 11.6 Hz, 2H), δ=3.69 (t, J = 11.92 Hz, 2H), δ=2.13 (m, 2H), δ=1.66 (m, 2H), δ=1.26-1.39 (m, 20H), δ=0.85 (m, 6H). ^{13}C NMR (151 MHz, $CDCl_3$) δ 166.54, 145.45, 133.81, 133.65, 129.83, 124.04, 123.60, 117.88, 57.61, 38.63, 31.77, 31.71, 29.90, 29.17, 29.15, 29.08, 28.88, 28.37, 27.01, 26.48, 22.61, 14.08. Anal. Calcd for $C_{32}H_{38}Br_2N_4O_2S_2$ (%): C 52.32, H 5.21, N 7.63, S 8.73. Found (%): C 52.35, H 5.24, N 7.64, S 8.71.

*Synthesis of **PTZBIBDT***

To a two-necked round-bottomed flask (25 mL) was added **Br_2-TZBI** (124.8 mg, 0.17mmol), **Sn_2-BDT** (153.5 mg, 0.17 mmol), anhydrous toluene (6 mL) and anhydrous DMF (1 mL) under argon. The mixture was purged with argon for 15 min. Then catalyst $Pd(PPh_3)_4$ (7 mg) was quickly added under a stream of argon, and the mixture was purged with argon for another 15 min. Subsequently, the reaction mixture was heated to reflux for 48 h with stirring. Then the reaction mixture was cooled to ambient temperature and precipitated into methanol. The solid was collected by filtration. After drying in the vacuum drying oven, the crude product was purified by Soxhlet extraction with methanol, acetone and hexane to remove oligomers and residual catalyst and the residual solid was dissolved in chloroform and

precipitated from methanol to get copolymer **PTZBIBDT** as a metallic golden solid (160 mg, yield = 82%). ^{1}H NMR (400MHz, $CDCl_3$): δ=8.13 (br, 2H), δ=7.82 (br, 2H), δ=7.38 (br, 4H), δ=6.97 (br, 2H), δ=4.81 (br, 2H), δ=3.73 (br, 2H), δ=2.92 (br, 4H), δ=2.18 (br, 2H), δ=1.72 (br, 2H), δ=1.27-1.48 (br, 38H), δ=0.85-0.99 (br, 18H). GPC (1,2,4-trichlorobenzene, 150 ^{o}C, polystyrene standard) M_n = 22.5 kDa, M_w = 56.7 kDa, PDI = 2.52. Anal. Calcd for $(C_{66}H_{80}N_4O_2S_6)_n$: C 68.71, H 6.99, N 4.86, S 16.68. Found (%): C 68.68, H 6.95, N 4.89, S 16.75.

3. Additional Figures and Tables

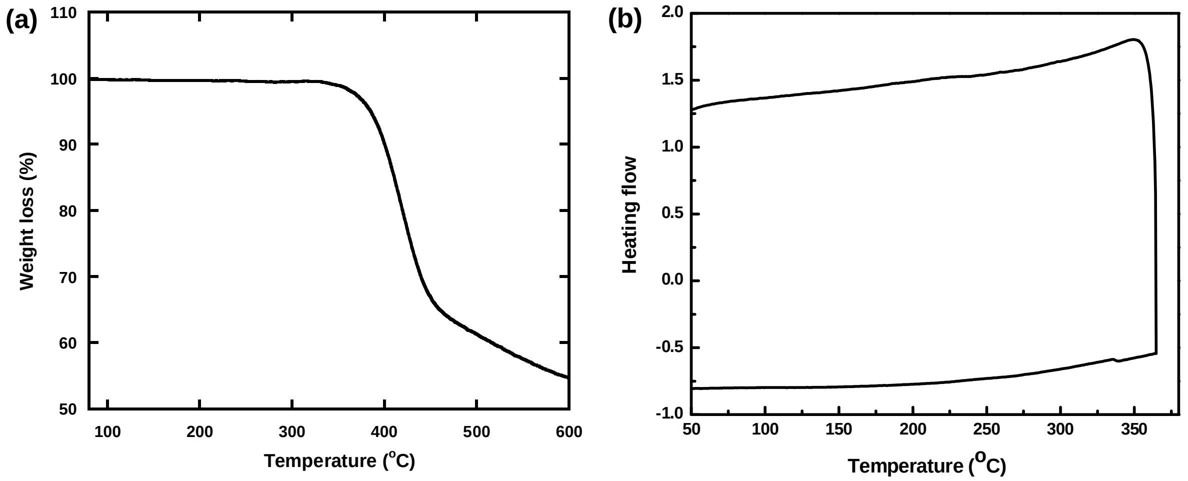

Figure S1. a) TGA plot of **PTZBIBDT** with a heating rate of 20 ^{o}C/min under inert atmosphere and b) Second heating and cooling DSC traces of **PTZBIBDT** with a heating rate of 10 ^{o}C min^{-1} and a cooling rate 20 ^{o}C min^{-1} under nitrogen.

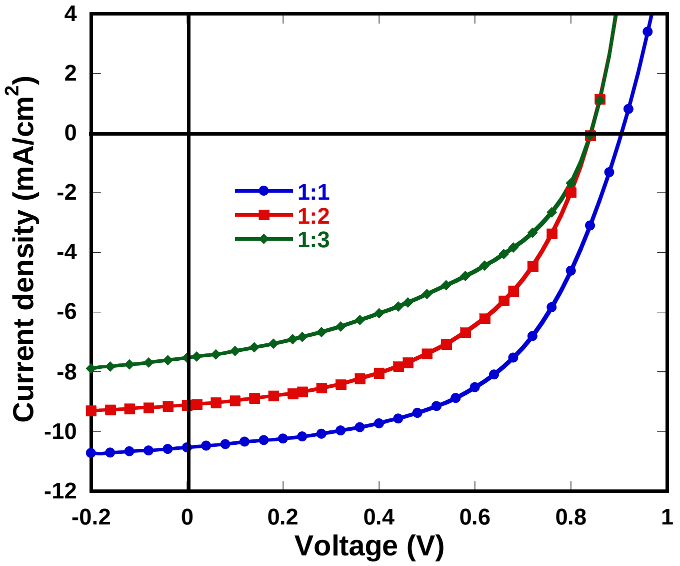

Figure S2. *J-V* curves of the PSCs based on different **PTZBIBDT**:$PC_{61}BM$ weight ratios in a device structure of ITO/PEDOT:PSS/active layer/Ca/Al.

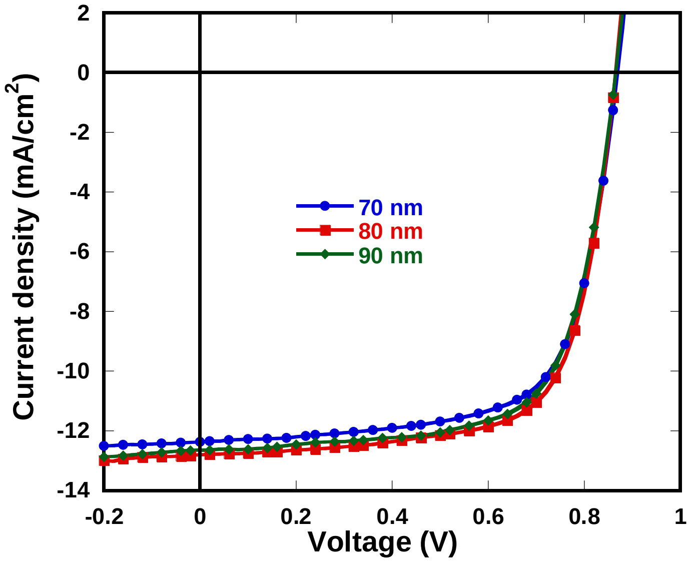

Figure S3. *J-V* curves of the PSCs with the active layer treated with 120 °C thermal annealing in a device structure of ITO/PEDOT:PSS/**PTZBIBDT**:$PC_{71}BM$ (w/w, 1:1)/Ca/Al with various active layer thickness.

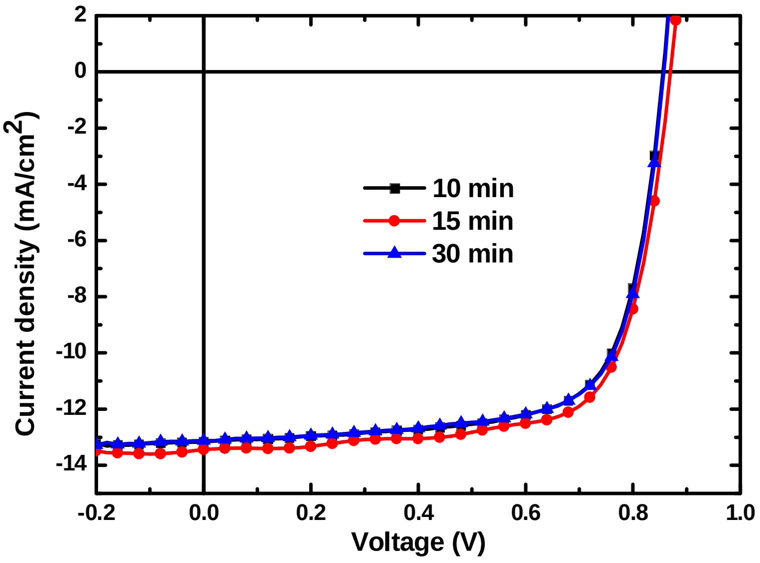

Figure S4. *J – V* curves of the PSCs with active layer thermally annealed at 120 °C for different time (10, 15 and 30 min). Device structure: ITO/PEDOT:PSS/**PTZBIBDT**:$PC_{71}BM$ (w/w, 1:1)/PFN/Al under the illumination of AM 1.5G, 100 mW cm^{-2}.

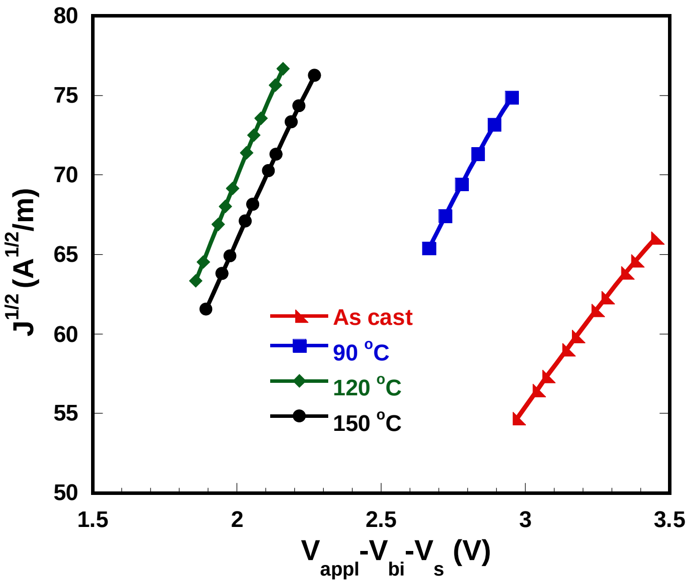

Figure S5. SCLC hole mobility measurement for **PTZBIBDT**:$PC_{71}BM$ = 1:1 blend treated with thermal annealing at different temperatures.

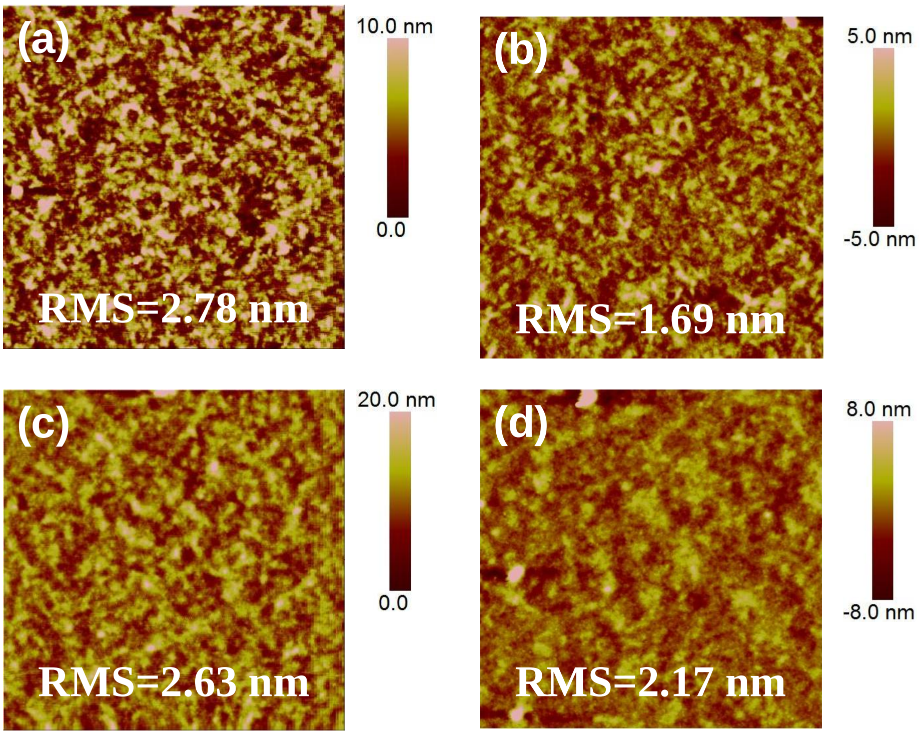

Figure S6. AFM height images (5 × 5 μm) of the **PTZBIBDT**:$PC_{71}BM$ (1:1, w/w) blend films: a) As-cast BHJ active layer; b) Active layer thermal annealed at 90 °C; c) Active layer thermal annealed at 120 °C; d) Active layer thermal annealed at 150 °C.

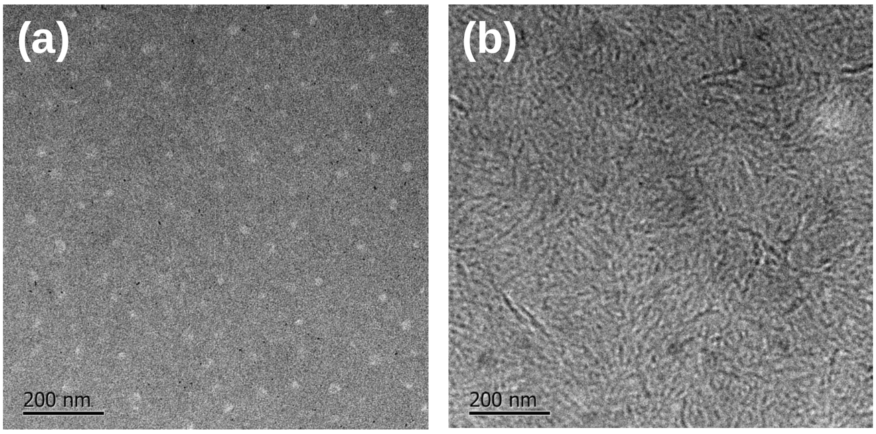

Figure S7. TEM images of the **PTZBIBDT**:$PC_{71}BM$ (1:1, w/w) blend films: a) As-cast BHJ active layer; b) Active layer thermal annealed at 120 °C.

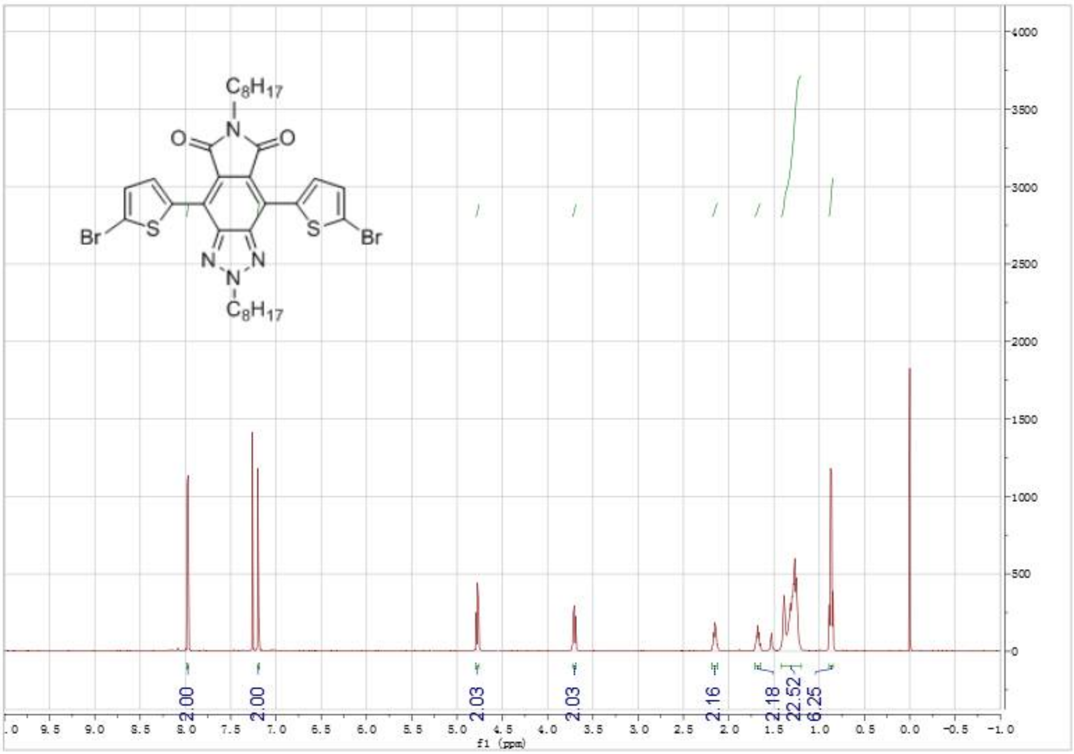

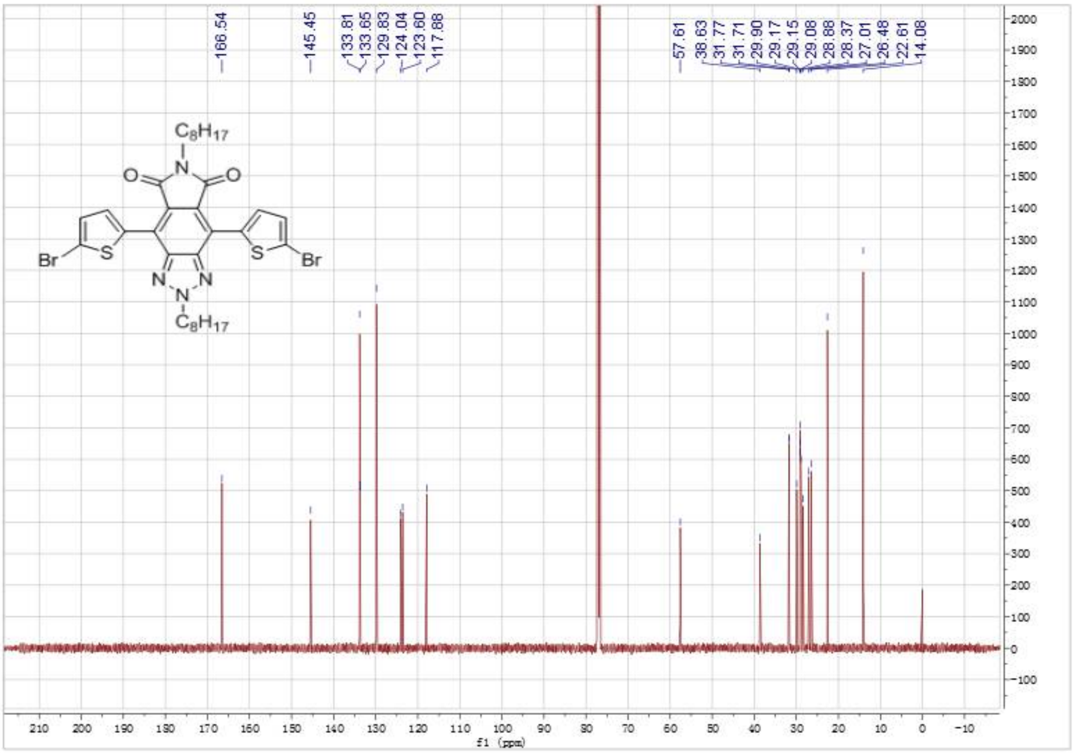

Figure S8. ^{1}H and ^{13}C NMR spectra of **Br_2-TZBI**.

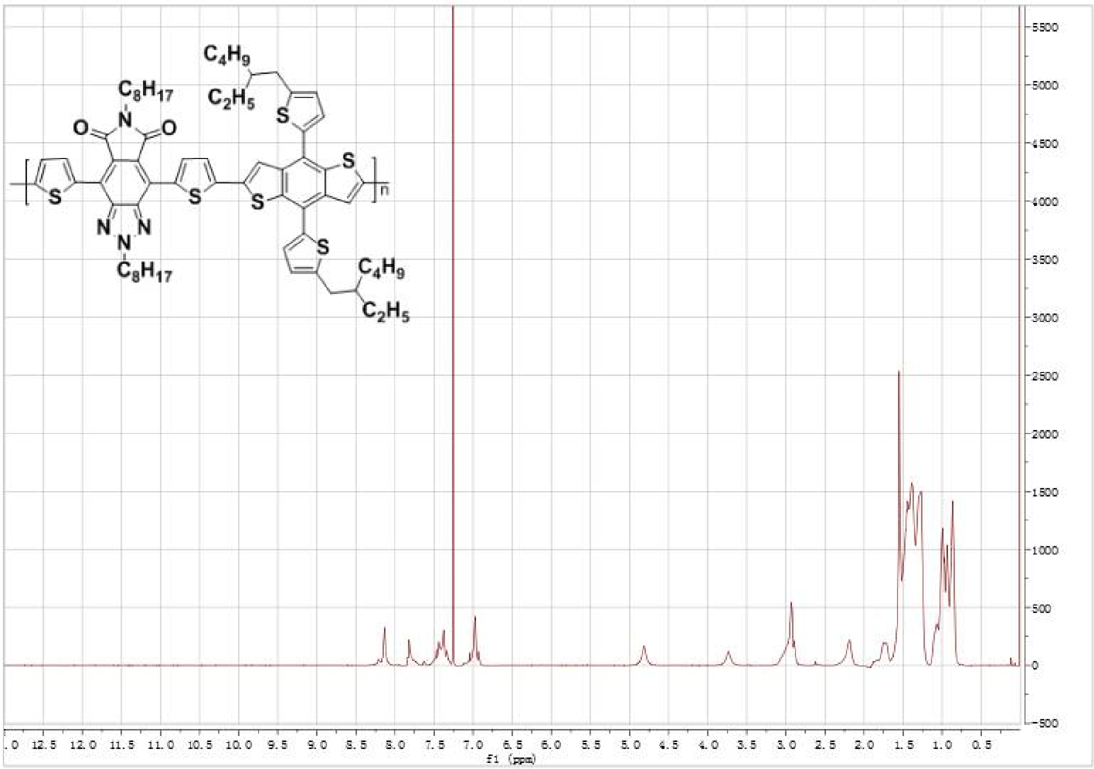

Figure S9. ^{1}H NMR spectrum of **PTZBIBDT**.

Table S1. Photovoltaic properties of the PSCs based on different **PTZBIBDT**:$PC_{61}BM$ weight ratios in a device struture of ITO/PEDOT:PSS/active layer/Ca/Al under the illumination of AM 1.5G, 100 mW cm^{-2}

**PTZBIBDT** /$PC_{61}BM$ (w/w)	V_{oc} (V)	J_{sc} (mA/cm^2)	FF (%)	Average PCE [a] (%)	PCE (%)
1:1	0.91	10.54	54.29	5.10 ± 0.08	5.18
1:2	0.84	9.11	50.45	3.60 ± 0.21	3.87
1:3	0.84	7.52	43.88	2.56 ± 0.18	2.78

a) The average PCE is calculated based on 10 devices.

Table S2. Photovoltaic properties of the PSCs with the active layer treated with 120 °C thermal annealing in a device struture of ITO/PEDOT:PSS/**PTZBIBDT**:$PC_{71}BM$ (w/w, 1:1)/Ca/Al with various active layer thickness under the illumination of AM 1.5G, 100 mW cm^{-2}

Thickness (nm)	V_{oc} (V)	J_{sc} (mA/cm^2)	FF (%)	Average PCE [a] (%)	PCE (%)
70	0.87	12.38	68.56	7.29 ± 0.06	7.37
80	0.87	12.81	69.79	7.57 ± 0.11	7.73
90	0.87	12.64	68.82	7.44 ± 0.08	7.52

a) The average PCE is calculated based on 10 devices.

Table S3. Photovoltaic properties of the PSCs with active layer thermally annealed at 120 °C for different time (10, 15 and 30 min). Device structure: ITO/PEDOT:PSS/**PTZBIBDT**:$PC_{71}BM$ (w/w, 1:1)/PFN/Al under the illumination of AM 1.5G, 100 mW cm^{-2}.

Annealing time (min)	V_{oc} (V)	J_{sc} (mA/cm^2)	FF (%)	Average PCE [a] (%)	PCE (%)
10	0.86	13.16	71.13	7.97 ± 0.04	8.02
15	0.87	13.50	71.09	8.27 ± 0.12	8.35
30	0.86	13.11	71.53	7.93 ± 0.08	8.04

a) The average PCE is calculated based on 10 devices.

[1] L. Y. Lan, Z. M. Chen, Y. C. Li, L. Ying, F. Huang, Y. Cao, *Polym. Chem.* **2015**, *6*, 7558.

[2] L. J. Huo, S. Q. Zhang, X. Guo, F. Xu, Y. F. Li, J. H. Hou, *Angew. Chem. Int. Ed.* **2011**, *50*, 9697.
